# Pestivirus Infections in Semi-Domesticated Eurasian Tundra Reindeer (*Rangifer tarandus tarandus*): A Retrospective Cross-Sectional Serological Study in Finnmark County, Norway

**DOI:** 10.3390/v12010029

**Published:** 2019-12-26

**Authors:** Carlos G. das Neves, Jonas Johansson Wensman, Ingebjørg Helena Nymo, Eystein Skjerve, Stefan Alenius, Morten Tryland

**Affiliations:** 1 Norwegian Veterinary Institute, N-0106 Oslo/N-9010 Tromsø, Norway; ingebjorg.nymo@vetinst.no; 2 Faculty of Health Sciences, UiT The Arctic University of Norway, N-9037 Tromsø, Norway; 3 Department of Clinical Sciences, Swedish University of Agricultural Sciences, P.O. Box 7054, SE-75007 Uppsala, Sweden; jonas.wensman@slu.se (J.J.W.); stefan.alenius@slu.se (S.A.); 4 Department of Arctic and Marine Biology, UiT The Arctic University of Norway, N-9037 Tromsø, Norway; 5 Centre for Epidemiology and Biostatistics, Faculty of Veterinary Sciences, Norwegian University of Life Sciences, N-0033 Oslo, Norway; eystein.skjerve@nmbu.no

**Keywords:** bvdv, epidemiology, reindeer, border disease virus, Norway

## Abstract

Members of the Pestivirus genus (family *Flaviviridae*) cause severe and economically important diseases in livestock. Serological studies have revealed the presence of pestiviruses in different cervid species, including wild and semi-domesticated Eurasian tundra reindeer. In this retrospective study, serum samples collected between 2006 and 2008 from 3339 semi-domesticated Eurasian reindeer from Finnmark County, Norway, were tested for anti-pestivirus antibodies using an enzyme linked immunosorbent assay (ELISA) and a subset of these by virus neutralization test (VNT). A seroprevalence of 12.5% was found, varying from 0% to 45% among different herding districts, and 20% in western Finnmark, as compared to 1.7% in eastern Finnmark. Seroprevalence increased with age. Pestivirus-specific RNA was not detected in any of the 225 serum samples tested by real-time RT-PCR. Based on VNT results, using a panel of one bovine viral diarrhea virus (BVDV) strain and two border disease virus (BDV) strains, the virus is most likely a reindeer-specific pestivirus closely related to BDV. A characterization of the causative virus and its pathogenic impact on reindeer populations, as well as its potential to infect other domestic and wild ruminants, should be further investigated.

## 1. Introduction 

Members of the *Pestivirus* genus, belonging to the family *Flaviviridae*, cause severe and economically important diseases in livestock [1] such as: bovine viral diarrhea virus (BVDV), causing bovine viral diarrhea (BVD) and mucosal disease (MD) in cattle; classical swine fever virus (CSFV)/hog cholera virus, causing classical swine fever in pigs; and border disease virus (BDV), causing border disease (BD) in sheep.

Several serological studies have revealed the presence of pestiviruses in a variety of free-ranging and captive wild cervid species [2,3]. The observed sero-prevalence has varied significantly between studies, geographic regions, cervid species, and proximity to other ruminant species known to harbor e.g., BVDV or BDV [4,5].

A few serological studies have been carried out in different reindeer subspecies and populations across the Arctic region, usually targeting BVDV but allowing for some cross-reactivity with other pestiviruses. In Sweden, several studies in semi-domesticated Eurasian tundra reindeer (*R. t. tarandus*) have revealed a prevalence ranging from 0 to 35% [5,6,7]. In Norway a study in several wild cervid species identified a prevalence of 4.2% in wild reindeer in southern Norway [8], while a study of 48 carcasses of emaciated semi-domesticated reindeer from Finnmark County, Norway, revealed a prevalence of 33% (BVDV virus neutralization test; VNT) [9]. A study of Svalbard reindeer (*R. t. platyrhynchus*) from the Arctic Archipelago of Svalbard in the 1990s revealed no seropositive animals [10].

Pestiviruses have never been isolated from wild or semi-domesticated reindeer. However, one isolate (Reindeer-1) was obtained from a reindeer that died with signs of severe diarrhea and anorexia at Duisburg Zoo in Germany in 1996 [11]. Phylogenetic studies have revealed that the strain was most closely related to BDV type 2 (BDV-2) strains isolated from German sheep in 1999 and 2000 [11,12].

In an experimental pestivirus infection (BVDV-1) of reindeer [13], the animals displayed clinical signs, such as serous and mucopurulent nasal discharge, bloody diarrhea, laminitis, and coronitis, thus indicating that this species is susceptible to BVDV.

Studies in the early 80s revealed that nearly 30% of the dairy herds in Norway had antibodies to BVDV [14]. An eradication program for cattle was initiated in 1992 with large-scale serological screenings focusing on PI animals and the enforcement of restrictions and culling. In November 2006, Norway was considered free of BVDV [15,16]. BD was identified in both sheep and goats in Norway in the early 1980s, but the disease was associated with BVDV strains rather than BDV strains, which have to our knowledge never been identified in Norway [14,17,18,19,20]. Even though the eradication program focused on cattle alone, infections from small ruminants have also not been reported since the 1990s. Studies in goats in Norway in the 1980s revealed low prevalence for pestivirus (3.6%) [21] and only a single PI goat kid was diagnosed [19].

Finnmark County hosts approximately 69% of the semi-domesticated reindeer in Norway, with an estimated population in 2017 of 147,500 reindeer (down from 170,000 reindeer in 2006 and 188,000 in 2008 at the time of sampling). Reindeer are kept under semi-nomadic husbandry conditions, free ranging but herded within well-defined husbandry districts, with seasonal migrations between summer pastures in coastal areas and winter pastures on the inland mountain plateau. Animals are usually gathered at least twice a year for tagging calves, anti-parasitic treatment, sorting, and slaughter.

Reindeer mortality in Finnmark County reached 37% among calves during the years of 2005/2006. Although predators accounted for the majority of mortality, 11% of calf mortalities were of unknown etiology [22]. Fecundity of semi-domesticated reindeer is difficult to evaluate as they usually give birth unattended, while scavengers quickly remove aborted materials. It is therefore difficult to assess the role of abortion or weak-born calves to calf mortality [23].

The goal of this study was to conduct a retrospective cross-sectional serological screening in selected reindeer husbandry districts, to address prevalence and to identify risk factors for becoming infected with pestivirus. It was also anticipated that a study of this nature could help identify which pestivirus species and/or strains are circulating among reindeer in Norway and if reindeer can serve as reservoir hosts for viruses known to cause disease in livestock.

## 2. Materials and Methods 

### 2.1. Sampling

In this retrospective study we used blood samples collected from reindeer (*n* = 3339) from 15 summer herding districts at four different slaughterhouses from 2004 to 2008 during the winter slaughtering periods in Finnmark County, Norway. Blood was centrifuged at 3500 rpm for 15 min and sera collected and stored at −20 °C until testing.

### 2.2. Animal Data

Information on gender, age, and carcass weight was collected from each herding district. Animals were grouped into two age classes: calves (≤1 year) and adults (>1 year).

For each reindeer herding district included in the study, information on total available reindeer pasture area, numbers of adults and calves, and mortality rates were obtained from the 2008 Reindeer Husbandry Authority Report [24]. Animal density, i.e., the number of animals per square kilometer of available reindeer summer pasture (n/km^2^), was calculated for each herding district. Districts with animal densities lower than the mean reindeer density for Finnmark (4.6/km^2^; range, 1.02–15.93/km^2^) were classified as low-density districts, whereas those with a density higher or equal to the mean were classified as high-density districts.

### 2.3. Enzyme-Linked Immunosorbent Assay (ELISA)

Antibodies to pestivirus were detected using a commercial blocking ELISA (SERELISA^®^ BVD p80 Ab Mono Blocking, Synbiotics, Lyon, France) that detects specific antibodies to a protein highly conserved in sequence between all strains of BVDV and BDV (p80/125 non-structural protein) [25,26]. Competition percentages and cut-off values were calculated according to the manufacturer’s instructions for testing small ruminant samples (i.e., sheep and goats). Samples were classified as positive if the competition percentage was greater than 40% and doubtful if it was between 20 and 40%.

To evaluate the kit’s performance with reindeer serum samples, we included in addition to the positive and negative bovine control sera supplied by the manufactures, fourteen reindeer sera previously classified as having pestivirus antibodies by VNT [9]. This ELISA kit has previously been used to detect antibodies against pestivirus in red deer (*Cervus elaphus*) [8], and other ruminant pestivirus ELISA kits with the same protein as antigen have been used to test other wild ruminant populations [5].

### 2.4. Virus Neutralization Test (VNT)

VNT was carried out to investigate to which ruminant virus the reindeer had been exposed. A panel of 30 samples, representative of six different geographical locations (three in eastern and three in western Finnmark), both age groups and both genders were selected. From each herding district, five samples were selected based on the ELISA results: two positive, one doubtful and two negatives. A panel of three different pestivirus strains (BVDV-1 strain NADL; BDV-1 strain 137/4, and BDV-2 strain Reindeer-1) were used to compare the capacity for neutralization of the selected reindeer test sera. Prior to incubation with the virus strains, the sera were heat-inactivated at 56 ºC for 30 min. The VNT was performed as described previously by Kautto and co-workers [5]. Samples were considered positive if neutralization was observed in at least one of two wells (replicates) at a dilution ≥ 1:4. BVDV negative serum was used as a negative control. Anti-BVDV polyclonal sera (VLA Weybridge) and ovine antisera raised against BDV-1 strain X818 and BDV-2 strain Reindeer-1 [12] were used as positive controls.

The neutralizing titers were calculated according to the Spearman–Kärber method [27] as the serum dilution necessary to neutralize the virus in 50% of the cell culture wells (effective dose 50%; ED_50_).

### 2.5. Real Time Reverse Transcriptase Polymerase Chain Reaction (Real-Time RT-PCR)

A total of 225 reindeer sera from all sampled districts, 15 samples per district, including animals with strong ELISA positive sera, as well as weak positive, doubtful, and negative sera were screened for the presence of pestivirus RNA by real-time reverse transcriptase polymerase chain reaction (real time RT-PCR). Whenever possible the following proportions, 2+1+12, between positive/weak positive, doubtful and negative animals were used when selecting samples per district, resulting in a total of 185 seronegative, 14 doubtful, and 26 seropositive samples being tested by real-time RT-PCR. Total RNA was extracted using QIAamp^®^ Viral RNA Mini Kit (Qiagen, Venlo, The Netherlands) according to the manufacturer’s instructions and eluted in a final volume of 40 µL. Reverse transcription was carried out with the iScript™ cDNA synthesis kit (random hexamer primers) from BioRad (Hercules, CA, USA) using the protocol supplied by the manufacturer [5]. For the following real-time PCR, we used the pan-pestivirus primers OPES13A: 5′-GCTAGCCATGCCCTTAGTAGGA -3′ and OPES14A: 5′- ATCAACTCCATGTGCCATTTACAGC -3′ at recommended primer concentrations [28] and the iQ™ SYBR^®^ Green Supermix from BioRad (CA, USA), in a total volume of 20 µL of PCR mix and 5 µL of template (cDNA) [5]. The PCR cycling conditions applied were as follows: primary denaturation at 95 ºC for 10 min, and 40 two-step amplification cycles at 95 ºC for 15 s and at 60 ºC for 1 min. Amplification products were verified for each run using a melting curve analysis of the gradient of temperature between 55 ºC and 95 ºC, after the final cycle. In addition, the sizes of the amplicons were verified by visualization in 1.5% agarose gel.

### 2.6. Statistical Analyses

Statistical analysis was carried out using Stata/SE 14 for Windows (Stata Corp., College Station, TX-USA). A non-parametric kernel density estimation of the probability density function for seroprevalence was performed to analyze the distribution of serological status between samples classified as positive, negative, or doubtful and to determine whether different cut-off calculations would have significant effects on results.

Weight was classified into 10 quantiles according to age and seroprevalence in order to assess the relationship between age and carcass weight and to verify that there were no major discrepancies.

Prevalence estimates were established using the survey commands of STATA; where district was the primary sampling unit for the model and data were stratified according to geographical area, carcass weight, and age class. Estimates were corrected using the following sample weighting procedure: 1/(N sampled reindeer/N total reindeer per district).

A multivariable logistic regression model was established using a backwards procedure adding initially all biological and ecological variables (gender, age, weight, geographical area, animal density, and year of sampling) and subsequently removing those with a *p*-value of the likelihood-ratio test >0.05. District was used as a cluster variable in the analyses and sample weighting was performed as for the prevalence estimates. The Hosmer and Lemeshow test for goodness of fit was carried out [29]. A classification table for sensitivity and specificity and a receiving operating characteristic (ROC) curve was calculated to assess the predictive qualities of the model.

ELISA and VNT titer results were categorized as follows: ELISA as positive if ≥ 40%, otherwise negative, and VNT as positive if titer ≥ 1:4, otherwise negative. The agreement between ELISA and VNT results was measured using the Cohen’s kappa (κ) test. A threshold of *p* = 0.05 was used when appropriate.

## 3. Results

### 3.1. Overall Results

**Figure 1:**
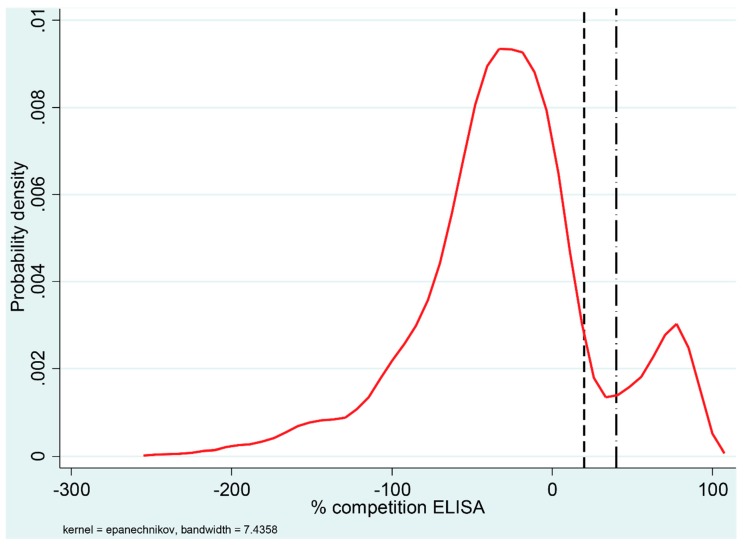
Distribution of percentage competition values for 3339 reindeer sera tested for antibodies against ruminant pestivirus using an ELISA. The density curve represents a kernel estimation of the probability density. The ELISA cut-off values are indicated in the graph by vertical lines; the dashed line (left) indicates a negative cut-off value of 20% and the dash-dot line (right) represents a positive cut-off value of 40%. Percentage competition values between 20% and 40% were considered doubtful.

The ELISA classified 418 of the 3339 reindeer samples as positive (12.5%) with an additional 89 samples classified as doubtful. The distribution of the percentage competition values of the ELISA results, using kernel density estimation, is shown in Figure 1. Positive and negative results formed two clearly distinguishable clusters and the positive results were concentrated above a competition percentage of 70%. 

**Figure 2:**
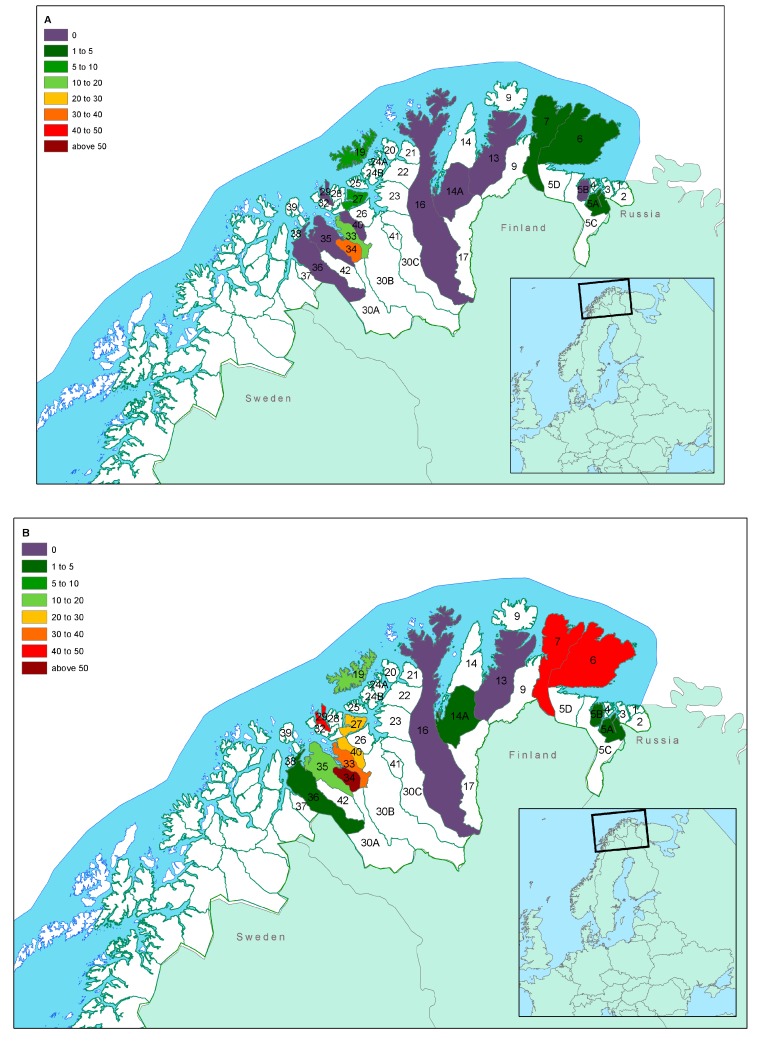
Seroprevalence of pestivirus infection according to herding district and age group (Map (**A**): calves; map (**B**): adults) in Finnmark County, Norway. Table 1 and Table 2 provide detailed information on age distribution within districts that should be considered when extrapolating the seroprevalence to the general population in each district. The border between western and eastern Finnmark is between districts 16 and 14A.

The overall seroprevalence by herding district level is shown in Table 1 and Figure 2A (calves) and Figure 2B (adults). Seroprevalence varied from 0% in district 13 to 44.8% in district 34. Table 1 further shows animal densities and the mean carcass weights according to district, which taken together are good indicators of asymmetries in sample composition. Seroprevalence (ELISA) was 1.7% in eastern Finnmark and 20.0% in western Finnmark. 

Extrapolation based on the inverse of the number of animals sampled per district divided by the total number of animals in the respective district showed that the samples included in this serosurvey were representative of 111,224 animals, accounting for 66.0% of the total reindeer population in Finnmark County. The overall seroprevalence was 12.5 to 15.2% before extrapolation and 8.2 to 10.2% after extrapolation. The seroprevalence ranged from 1.9 to 2.8% in eastern Finnmark and from 18.1 to 21.2% in western Finnmark.

### 3.2. Age Classification and Weight Classes

Seroprevalence increased from the lowest weight class (quantile 1; seroprevalence = 3.7%) to the highest weight class (quantile 10; seroprevalence = 21.4%). Table 2 shows the relationships between carcass weight and seroprevalence when carcass weight was stratified into 10 quantiles.

### 3.3. Factors Affecting Seroprevalence

Carcass weight was positively correlated with seroprevalence in calves and adults up to 35 kg, irrespective of gender or geographical location (Table 2). No interactions were detected between carcass weight and any of the other variables.

The logistic regression model (Table 3) showed that geographic location, age and gender affected seroprevalence to differing extents. Age (odds ratio = 5.57, CI [2668–11,629]) clearly affected seroprevalence with adult animals having a much higher chance of being seropositive than calves. Geographic location had a minimal effect (odds ratio = 0.084), with animals in western Finnmark having a slightly higher chance of being positive. Concerning gender there seemed to be a slightly higher chance of males being positive than females (odds ratio = 0.472). Differences in seroprevalence between variations in animal density by district or year of sampling were not statistically significant (*p* > 0.05). 

None of the additional tests indicated that the model did not fit the data. The Hosmer and Lemeshow goodness of fit test had a chi-square value of 0.46 and a *p* value of 0.977 (in this instance, the *p* value is significant if it is greater than 0.05), implying that the model’s estimates fitted the data at an acceptable level. 

A classification table was compiled to determine the accuracy of the logistic regression model. For a cut-off value of 50% probability (*p* = 0.5), the model correctly predicted the classification of 88.4% of the samples. The area under the ROC curve was 0.81.

### 3.4. Virus Neutralization Test (VNT)

VNT results are summarized in Table 4, in which the ED_50_ values are presented as the neutralizing titer that corresponded with the respective log_2_ ED_50_. VNT showed that samples negative according to the ELISA were unable to neutralize any of the ruminant pestiviruses, thus being in concordance with the ELISA results for these samples. Doubtful samples had on average low titers for BDV1 and BDV2 and none against BVDV1. All samples that were positive according to the ELISA had higher neutralization titers for BDV1 (average 1:126) and BDV2 (average 1:131) than BVDV1 (average 1:19). Titers against BDV1 and BDV2 were usually very close to each other (within 1 log and non-statistically relevant), with the exception of district 16 where neutralizing titers against BDV1 were not observed. 

Titers against BVDV1 were consistently much lower in every district than those observed for BDV1 and BDV2. No serum sample neutralized BVDV1 at a higher dilution than it neutralized the BDV1 and BDV2.

Statistical analysis of the agreement between ELISA and VNT showed a good correlation between the two tests, where samples with increasing competition ability (ELISA) also had increasing VNT titers. The best correlation between the ELISA and the VNT was found when BDV2 was used in the VNT (κ = 0.580 for ELISA and BVDV; κ = 0.723 for ELISA and BDV1; κ = 0.862 for ELISA and BDV2).

### 3.5. Realtime RT-PCR

None of the reindeer serum samples selected for real time RT-PCR were positive. Pestivirus positive controls were positive, with an amplicon of the expected size of around 295 base pairs confirmed by electrophoresis.

## 4. Discussion

The presence of seropositive animals in several districts across eastern and western Finnmark confirmed that a pestivirus is enzootic among semi-domesticated reindeer in Norway. VNT results seem to indicate that the virus is more closely related to BDV than to BVDV1.

Serum samples classified as positive and negative in the ELISA comprised two clearly separated clusters, whereas the probability of a sample being doubtful (i.e., 20–40% competition) was low (Figure 1). Furthermore, all samples classified as negative in the ELISA and subsequently tested in the VNT failed to neutralize the actual virus tested. Thus, we believe that the ELISA cut-off values chosen with this serological kit can be applied when testing reindeer samples. The viral envelope non-structural protein p80 is highly conserved between the different BVDV strains and represents one of the most important markers of cp BVDV [25,26].

The seroprevalence in this study ranged from 0 to 51%, with a mean value of 12 to 15%, and increased with age. This is in line with previous studies, although mean values of prevalence vary considerably between studies. Studies in Sweden identified a prevalence of 35% (5], while previous studies in Norway found a prevalence between 4% [8] and 33% [9]. Differences in analytical methods, geographic regions, and selection criteria for test animals from the herds are some factors that might help explain these differences. It is, however, beyond doubt that pestiviruses are circulating continuously in most tested reindeer populations.

Figure 2B shows two clusters of relatively high prevalence in adults. The cluster around districts 33, 34, and 40 shows a pattern already described for alphaherpesvirus infections [30], in a study that tested the same animals for cervid herpesvirus 2. While these are areas with a higher number of livestock, as compared to districts in eastern Finnmark, the indications that both alphaherpes- and pestiviruses circulating in the reindeer populations are reindeer-specific, suggest that the presence of domestic animals cannot explain the high prevalence in reindeer. Whether this could be the result of husbandry practices, reduced area for the animals to graze (temporary high-density episodes) or presence of other predisposing factors that may increase infection with these two viruses, remains unknown and should be a target for further investigations.

Carcass weight was an important risk factor for seropositivity. The mean weight value for each quantile (Table 2) can be used to correctly interpret seroprevalence at district level (Table 1). Our method of expressing the results as a cross interpretation between weight and seroprevalence avoids misclassification of age. This could have affected the data from 275 animals in this study and resulted in misinterpretation of the results (as shown by some discrepancies in the weight quantiles shown in Table 2, i.e., calves in quantiles 5 to 10 and adults in quantiles 1 to 4).

The strong correlation between seroprevalence and weight/age identified in this study is supported by previous studies [5,8] and supports previous findings of life-long immunity to pestivirus infection. Most likely, the source of infection are PI animals.

Geographic origin (district) and gender seemed to have only a small impact on the risk of infection. We find no compelling argument to explain the slightly higher likelihood of males becoming infected as compared to females, since there is no special difference in the management of males and females that could account for this difference. We thus argue that this association might be the result of a possible bias in the sampling between genders. In fact, several studies of pestivirus infections in wildlife have tried to assess the effect of gender, often obtaining weak or non-significant associations or sometimes-contradictory associations [31].

VNT results indicate that the virus circulating in reindeer is more closely related to BDV1 and BDV2 than BVDV1. Since BVDV was eradicated from domestic animals in Norway in 2006 [15], and BDV has never been reported in Norway [17], the VNT results might therefore indicate the circulation of an unknown virus more closely related to BDV. The relatively low titers against the BDV1 and BDV2 could indicate reduced affinity (i.e., heterologous viruses), but may also suggest a reduction of circulating antibodies over time. The lack of new outbreaks of BVDV1 in cattle in Norway also supports the conclusion that it is unlikely that reindeer would be harboring this virus (as confirmed by low titers in our VNT). Reindeer have sporadic contact with both cattle and small ruminants and one could presume if BVDV1 were circulating in reindeer, it would have been detected during routine screenings of livestock.

Real-time RT-PCR failed to identify any sample positive for viral RNA. Detection of viral RNA is only possible either from PI animals or from transiently infected animals sampled during the period of viremia, which in cervids may last less than 5 days [32]. The lack of detection of PI animals by RT-PCR is not surprising as these animals usually represent a very small fraction of any given host population [33,34]. A study in Sweden including more than 276 reindeer serum samples from districts with high pestivirus seroprevalence also failed to detect viral RNA by real-time RT-PCR [5]. 

The high seroprevalence in adult animals likely represents continuous episodes of transmission. This requires the presence of PI animals, generated by transplacental infection from a transiently infected mother. Since the fetus must be infected during early pregnancy to become PI, and since this period coincides with the peak of winter when domestic animals are housed, the chance of domestic animals contributing to the maintenance of the infection cycle in reindeer is small or non-existent [5]. This is further supported by the absence of pestivirus infections in domestic ruminants in Norway.

Infections known to be involved in abortion or reduced survival of newborns, such as herpes or pestivirus infections, should be further studied to gain a better understanding of factors affecting mortality and reproductive success. The identification of abortion related to pestiviruses or the mortality of young animals due to mucosal disease—both often described in cattle associated with BVDV [35]—is however difficult, given that aborted calves and dead animals are quickly removed by scavengers.

Another important factor that remains unknown at this point is the potential pathogenicity of a reindeer-specific pestivirus to other ruminant species (wild or domesticated). PI sheep with BDV1 have been shown to transmit the virus to seronegative calves and adult cattle [36,37]. Given the relatively high seroprevalence among reindeer and the possibility of contact with cattle, goats or sheep, it might be that this transmission potential is reduced, or that the virus in question produces mild or no clinical signs in other species.

In conclusion, the present results confirm the circulation of a hitherto unknown pestivirus in the semi-domesticated reindeer population of northern Norway that seems to be serologically closely related to the BDV genotype group 2. Characterization of the causative virus, its infection biology and pathogenic impact in the reindeer populations, as well as its potential to infect other domestic and wild ruminants, should be further investigated.

## Figures and Tables

**Table 1:** Distribution of ruminant pestivirus seroprevalence according to reindeer herding district in Finnmark County, Norway.

Area	District	Density	Weight	Age	Seroprevalence		
		(n/km^2^)	Mean	(C/A) ^a^	N +/total *(Doubt)* ^b^	(%) ^c^	95% CI ^d^
East	5A	1.0	20.3	90/33	1/185 *(1)*	0.5–1.1	[0–2.6]
	5B	4.2	--- ^e^	20/53	1/73 *(1)*	1.4–2.7	[0–6.5]
	6	2.1	19.5	110/5	4/119 *(1)*	3.4–4.2	[0.1–7.8]
	7	1.4	24.7	45/23	12/73 *(2)*	16.4–19.2	[7.9–28.3]
	13	4.4	21.8	175/73	0/256 (2)	0–0.8	[0–1.9]
	14A	4.3	31.8	10/56	1/66 *(0)*	1.5	[0–4.5]
	16	7.9	24.2	150/426	4/588 *(7)*	0.7–1.9	[0–3.0]
**E subtotal**		**2.9**	**23.5**	**600/699**	**23/1360 *(14)***	**1.7–2.7**	**[1.0–3.6]**
West	19	4.7		245/38	19/283 *(5)*	6.7–8.5	[3.8–11.7]
	27	14.9	20.9	211/241	74/452 *(13)*	16.4–19.2	[13.0–22.9]
	29	6.0	35.9	1/42	18/43 *(1)*	41.9–44.2	[26.9–59.2]
	33	11.2	23.9	10/145	71/234 *(15)*	30.3–36.8	[24.4–42.9]
	34	11.5	23.7	63/163	162/362 *(25)*	44.8–51.7	[39.6–56.8]
	35	5.5	19.2	194/28	4/224 *(5)*	1.8–4.0	[0–6.6]
	36	3.9	25.9	49/131	5/202 *(0)*	2.5	[0.3–4.6]
	40	15.9	24.5	2/177	42/179 *(11)*	23.5–29.6	[17.2–36.3]
**W subtotal**		**6.6**	**22.9**	**775/965**	**395/1979 *(75)***	**20.0–23.7**	**[18.2–25.6]**
**Total**		**4.1**	**23.2**	**1375/1634**	**418/3339 *(89)***	**12.5–15.2**	**[11.4–16.4]**

**^a^** Age distribution between calves (≤1 year) and adults (>1 year). Information on age was only available for 3009 animals. **^b^** Bracketed values represent the number of animals classified as doubtful after retesting. **^c^** Seroprevalence is presented as an interval between % calculated from ELISA positive animals only and % including all doubtful results as positives. **^d^** Confidence intervals calculated for the maximum interval when animals classified as doubtful were included as if they were positive. **^e^** No information regarding weight was available for animals sampled in district 5B.

**Table 2:** Distribution of seroprevalence in 10 carcass weight quantiles and classification of age.

Carcass Weight			Number of Animals		Seroprevalence		
Quantile	Mean (kg)	[Min–Max] (kg)	Calves	Adults	N	%	95% CI
1	13.9	[9.2–15.7]	352	5	22	6.2	[3.7–8.7]
2	16.8	[15.7–17.7]	255	18	13	4.8	[2.2–7.3]
3	18.7	[17.7–19.5]	232	34	13	4.9	[2.3–7.5]
4	20.7	[19.5–21.2]	195	95	18	6.2	[3.4–9.0]
5	22.1	[21.2–22.9]	142	182	36	11.1	[7.7–14.5]
6	23.8	[22.9–24.8]	108	236	47	13.7	[10.0–17.3]
7	25.9	[24.8–27.0]	65	281	55	16.0	[12.1–20.0]
8	27.9	[27.0–29.0]	15	269	59	20.8	[16.0–25.5]
9	30.5	[29.0–32.3]	11	247	41	15.9	[11.4–20.4]
10	38.9	[32.3–69.7]	1	265	45	16.9	[12.4–21.4]

Data from 3008 animals were used since data on weight and age were not available for all animals. Animals were classified as calves (≤1 year old) or adults (>1 year old).

**Table 3:** Logistic regression model for risk factors associated with becoming infected with pestivirus.

Pestivirus Seroprevalence	Odds Ratio	Robust SE	z	P>|z|	95% CI
Region (west to east)	0.084	0.062	−3.36	0.001	[0.020–0.356]
Age (calves to adults)	5.570	2.092	4.57	<0.0001	[2.668–11.629]
Gender (male to female)	0.472	0.167	−2.12	0.034	[0.236–0.944]
Constant	0.093	0.038	−5.82	<0.0001	[0.042–0.207]
No. of observations	3009				
The standard error was adjusted for 15 clusters over districts. *p* values < 0.05 were regarded as statistically significant. Non-significant interactions were removed from the model					

**Table 4:** Virus neutralization test of reindeer sera samples for ruminant pestiviruses.

		ELISA	VNT ($\bar{x}$ Titer)		
Region	District	$\bar{x}$ % Compet.	BVDV NADL	BDV1 137/4	BDV2 Reindeer-1
East	5	64.8	6	68	130
	7	78.4	12	192	96
	16	76.2	6	6	192
West	19	84.2	66	264	132
	34	85.5	6	160	144
	40	79.5	18	128	160
	**TOTAL (positives)**	**78.1**	**19**	**136**	**142**
	Doubtful (all districts)	33.5	7	33	24
	Negative (all districts)	−68.0	<4	<4	<4

A panel of 30 samples representative of different geographical locations. Both age groups and both genders were selected. From each district, five samples were selected based on the ELISA results: two seropositive, one doubtful, and two seronegative. The neutralizing titers were calculated according to the Spearman–Kärber method as the serum dilution necessary to neutralize the virus in 50% of the wells (effective dose 50%; ED50). Average titers were calculated per district.

## References

[1] Murphy F.A., Gibbs E.P., Horzinek M.C., Studdert M.J., Murphy F.A., Gibbs E.P.J., Horzinek M.C., Studdert M.J. (1999). Laboratory Diagnosis of Viral Diseases. Veterinary Virology.

[2] Vilcek S., Nettleton P.F. (2006). Pestiviruses in wild animals. Vet. Microbiol..

[3] Ridpath J.F., Neill J.D. (2016). Challenges in Identifying and Determining the Impacts of Infection with Pestiviruses on the Herd Health of Free Ranging Cervid Populations. Front. Microbiol..

[4] Larska M. (2015). Pestivirus infection in reindeer. Front. Microbiol..

[5] Kautto A.H., Alenius S., Mossing T., Becher P., Belak S., Larska M. (2012). Pestivirus and alphaherpesvirus infections in Swedish reindeer (*Rangifer tarandus tarandus* L.). Vet. Microbiol..

[6] Rehbinder C., Nordkvist M. (1985). A suspected virus infection of the oral mucosa in Swedish reindeer (*Rangifer tarandus*). Rangifer.

[7] Rehbinder C., Belak K., Nordkvist M. (1992). A serological, retrospective study in reindeer on five different viruses. Rangifer.

[8] Lillehaug A., Vikoren T., Larsen I.L., Akerstedt J., Tharaldsen J., Handeland K. (2003). Antibodies to ruminant alpha-herpesviruses and pestiviruses in Norwegian cervids. J. Wildl. Dis..

[9] Tryland M., Mørk T., Ryeng K.A., Sørensen K.K. (2005). Evidence of parapox-, alphaherpes- and pestivirus infections in carcasses of semi-domesticated reindeer (*Rangifer tarandus tarandus*) from Finnmark, Norway. Rangifer.

[10] Stuen S., Krogsrud J., Hyllseth B., Tyler N.J.C. (1993). Serosurvey of three virus infections in reindeer in northern Norway and Svalbard. Rangifer.

[11] Becher P., Orlich M., Kosmidou A., Konig M., Baroth M., Thiel H.J. (1999). Genetic diversity of pestiviruses: Identification of novel groups and implications for classification. Virology.

[12] Avalos-Ramirez R., Orlich M., Thiel H.J., Becher P. (2001). Evidence for the presence of two novel pestivirus species. Virology.

[13] Morton J., Evermann J.F., Dieterich R.A. (1990). Experimental infection of reindeer with bovine viral diarrhea virus. Rangifer.

[14] Loken T., Krogsrud J., Larsen I.L. (1991). Pestivirus infections in Norway. Serological investigations in cattle, sheep and pigs. Acta Vet. Scand..

[15] Kampen A.H., Åkerstedt J., Gudmundsson S., Hopp P., Grøneng G., Nyberg O. (2007). The Surveillance and Control Programme for Bovine Virus Diarrhoea (BVD) in Norway.

[16] Loken T., Nyberg O. (2013). Eradication of BVDV in cattle: The Norwegian project. Vet. Rec..

[17] Loken T. (1992). Pestivirus infections in ruminants in Norway. Revue Sci. Tech.-Off. Int. Épizoot..

[18] Loken T., Barlow R.M. (1981). Border disease in Norway. Acta Vet. Scand..

[19] Loken T., Bjerkas I., Hyllseth B. (1982). Border disease in goats in Norway. Res. Vet. Sci..

[20] Loken T., Hyllseth B., Larsen H.J. (1982). Border disease in Norway. Serological examination of affected sheep flocks. Acta Vet. Scand..

[21] Loken T. (1990). Pestivirus infections in Norway. Epidemiological studies in goats. J. Comp. Pathol..

[22] Ressursregnskap for Reindriftsnaeringen.

[23] Tveraa T., Fauchald P., Henaug C., Yoccoz N.G. (2003). An examination of a compensatory relationship between food limitation and predation in semi-domesticated reindeer. Oecologia.

[24] (2008). Ressursregnskap for Reindriftsnaeringen.

[25] Collett M.S. (1992). Molecular genetics of pestiviruses. Comp. Immunol. Microbiol. Infect. Dis..

[26] Deregt D., Masri S.A., Cho H.J., Bielefeldt O.H. (1990). Monoclonal antibodies to the p80/125 gp53 proteins of bovine viral diarrhea virus: Their potential use as diagnostic reagents. Can. J. Vet. Res..

[27] Thrusfield M., Thrusfield M. (1986). Serological epidemiology. Veterinary Epidemiology.

[28] Elvander M., Baule C., Persson M., Egyed L., Ballagi-Pordany A., Belak S., Alenius S. (1998). An experimental study of a concurrent primary infection with bovine respiratory syncytial virus (BRSV) and bovine viral diarrhoea virus (BVDV) in calves. Acta Vet. Scand..

[29] Hosmer D.W., Lemeshow S. (1989). Applied Logistic Regression.

[30] das Neves C.G., Thiry J., Skjerve E., Yoccoz N.G., Rimstad E., Thiry E., Tryland M. (2009). Alphaherpesvirus infections in semidomesticated reindeer: A cross-sectional serological study. Vet. Microbiol..

[31] Pioz M., Loison A., Gibert P., Dubray D., Menaut P., Le Tallec B., Artois M., Gilot-Fromont E. (2007). Transmission of a pestivirus infection in a population of Pyrenean chamois. Vet. Microbiol..

[32] Ridpath J.F., Mark C.S., Chase C.C., Ridpath A.C., Neill J.D. (2007). Febrile response and decrease in circulating lymphocytes following acute infection of white-tailed deer fawns with either a BVDV1 or a BVDV2 strain. J. Wildl. Dis..

[33] Hessman B.E., Fulton R.W., Sjeklocha D.B., Murphy T.A., Ridpath J.F., Payton M.E. (2009). Evaluation of economic effects and the health and performance of the general cattle population after exposure to cattle persistently infected with bovine viral diarrhea virus in a starter feedlot. Am. J. Vet. Res..

[34] Loneragan G.H., Thomson D.U., Montgomery D.L., Mason G.L., Larson R.L. (2005). Prevalence, outcome, and health consequences associated with persistent infection with bovine viral diarrhea virus in feedlot cattle. J. Am. Vet. Med. Assoc..

[35] Schweizer M., Peterhans E. (2014). Pestiviruses. Annu. Rev. Anim. Biosci..

[36] Braun U., Bachofen C., Buchi R., Hassig M., Peterhans E. (2013). Infection of cattle with Border disease virus by sheep on communal alpine pastures. Schweiz. Arch. Tierheilkd..

[37] Braun U., Reichle S.F., Reichert C., Hassig M., Stalder H.P., Bachofen C., Peterhans E. (2014). Sheep persistently infected with Border disease readily transmit virus to calves seronegative to BVD virus. Vet. Microbiol..

